# Consolidating emerging evidence surrounding HIVST and HIVSS: a rapid systematic mapping protocol

**DOI:** 10.1186/s13643-017-0452-4

**Published:** 2017-04-05

**Authors:** T. Charles Witzel, Peter Weatherburn, Fiona M. Burns, Cheryl C. Johnson, Carmen Figueroa, Alison J. Rodger

**Affiliations:** 1 Sigma Research, Department of Social and Environmental Health Research, Faculty of Public Health and Policy, London School of Hygiene and Tropical Medicine, 15-17 Tavistock Place, London, WC1H 9SH UK; 2 Research Department of Infection & Population Health, UCL and Royal Free London NHS Foundation Trust, London, UK; 3 Department of HIV, World Health Organization, Geneva, Switzerland

## Abstract

**Background:**

HIV self-testing (HIVST) is becoming popular with policy makers and commissioners globally, with a key aim of expanding access through reducing barriers to testing for individuals at risk of HIV infection. HIV self-sampling (HIVSS) was available previously to self-testing but was confined mainly to the USA and the UK. It remains to be seen whether the momentum behind HIVST will also energise efforts to expand HIVSS. Recent years have seen a rapid growth in the type of evidence related to these interventions as well as several systematic reviews. The vast majority of this evidence relates to acceptability as well as values and preferences, although new types of evidence are emerging. This systematic map aims to consolidate all emerging evidence related to HIVST and HIVSS to respond to this rapidly changing area.

**Methods:**

We will systematically search databases and the abstracts of five conferences from 2006 to the present date, with monthly-automated database searches. Searches will combine key terms relating to HIV (e.g. HIV, AIDS, human immune-deficiency syndrome) with terms related to self-testing (e.g. home-test, self-test, mail-test, home dried blood spot test). Abstracts will be reviewed against inclusion criteria in duplicate. Data will be manually extracted through a standard form and then entered to an open access relational map (HIVST.org). When new and sufficient evidence emerges which addresses existing knowledge gaps, we will complete a review on a relevant topic.

**Discussion:**

This innovative approach will allow rapid cataloguing, documenting and dissemination of new evidence and key findings as they emerge into the public domain.

**Systematic review registration:**

This protocol has not been registered with PROSPERO as they do not register systematic maps.

**Electronic supplementary material:**

The online version of this article (doi:10.1186/s13643-017-0452-4) contains supplementary material, which is available to authorized users.

## Background

HIV self-testing (HIVST) as an intervention and a potential HIV prevention tool is gaining popularity amongst policy makers globally, as well as amongst key populations affected by HIV. While previously HIVST was uncommon and generally confined to individuals working in healthcare settings informally testing themselves [1], changes in attitudes towards HIV infection brought about by the availability of highly effective HIV treatment have led many countries to introduce legal and policy changes to allow HIVST kits to be licensed and distributed formally [2].

In some settings, HIV self-sampling (HIVSS) gained popularity before HIVST became available [2]. HIVSS was approved in the USA by the Food and Drug Administration in 1996 in response to a desire amongst clinicians and service providers for greater patient autonomy in healthcare. HIVSS harnesses the convenient and private nature of HIVST while also providing support in the form of a laboratory to process the sample and a trained counsellor who could provide results in an effort to counter some of the concerns around self-harm, adverse events and issues with user error [2].

The development of and wide availability of low-cost rapid diagnostic tests (RDTs) have also made it possible to expand testing within and beyond facilities by using community-based approaches, which are increasingly used in HIV testing in low-, middle- and high-income settings [3, 4]. The wider availability and lower cost of anti-retroviral therapy (ART) alongside evidence indicating that early diagnosis and immediate treatment initiation improve patient health outcomes, and enable HIV-positive patients to achieve virological suppression which can then prevent onwards transmission, have strengthened the policy argument for the expansion of testing [5–7].

HIVST in particular is now considered to have an important role as part of the expansion of standard HIV testing services and is congruent with widespread shifts in western health systems which increasingly emphasise patient self-management and autonomy [8].

The last 5 years have seen a rapid emergence of published data and corresponding reviews synthesising evidence around HIVSS and HIVST [9–13]. Reflecting the distribution of evidence at the time, the first and most far ranging of these reviews [10] relied substantially on relatively few papers, mainly from the USA.

Since 2013, there has been an increasing flow of evidence about self-sampling and self-testing from a wider range of countries including Australia [14–16], Brazil [17], China [18, 19], the USA [20–22], the UK [23–26], Malawi [27–30] and Lesotho [31]. There remain very little European implementation-based evidence and evidence related to patient experience of HIVST. Aside from exploratory surveys and recent reviews [9–13, 32] in which the majority of included data on the perceptions of values and preferences around HIVST arise from studies of gay, bisexual or other men who have sex with men (MSM), almost exclusively in industrialised countries (and still disproportionately from the USA as well as Australia), there has also been a wide range of opinion pieces broadly supportive of HIVST [2, 33–35].

The vast majority of this research, and hence the focus of all the reviews, has been the acceptability of HIVST, HIVSS and the values and preferences that inform the responses of key populations (MSM, transgender people, sex workers and injection drug users) to these emerging technologies. There can be little doubt that many individuals find the notion of HIVST to be acceptable and that it is feasible to deliver HIVST services in a range of settings in high-income countries. To quote the most recent, [13], “in general, MSM were interested in HIVST because of its convenient and private nature. However, they had concerns about the lack of counselling, possible user error and accuracy” (of test results).

Recognising that the limited evidence base had already been repeatedly reviewed, we will undertake a systematic mapping exercise to formalise the literature searching process behind HIVST.org, an online centre for public health research, documentation and policies regarding HIV self-testing. A redesign of this website will create a searchable relational database where all literature on HIVSS and HIVST globally can be consolidated.

Systematic mapping is a process of collecting, collating and describing the parameters of the existing literature on a particular topic [36]. This systematic map will focus on HIV testing modalities. This tool will offer policy makers, practitioners and researchers an explicit and transparent means of identifying narrower policy and practice-relevant review questions. It also enables the contextualisation of in-depth systematic literature reviews within the broader literature and identification of gaps in the evidence base. Systematic mapping was originally developed by the EPPI-Centre at the Social Science Research Unit, then part of the Institute of Education at the University of London [37].

## Aims and objectives

### Aim

The aim of this paper is to consolidate emerging evidence related to HIVST and HIVSS.

### Objectives

- To monitor and catalogue changes in published evidence as it emerges
- To collate evidence describing social and contextual factors shaping HIV testing practices, especially barriers to testing, perceptions of the acceptability of self-testing and self-sampling relative to other testing approaches and associated issues including management of psychological effects and subsequent pathways to care
- To document impacts from the expansion of HIVST as the technology continues to gain popularity

## Methods

The Preferred Reporting Items for Systematic Review and Meta-Analysis Protocols (PRISMA-P) were used to prepare this protocol [see Additional file 1].

### Search strategy

Published studies will be captured through a variety of standard searches which were trialled and refined throughout January 2016. Going forward searches will be automated to provide updates on the first of every month. This will enable us to capture all new articles published in that month and monitor changes in the published literature in real time. Searches will cover from January 1, 2006, to the present date, reflecting changes in the nature of HIV infection following widespread availability of anti-retroviral therapy and the emerging imperative to expand HIV testing [34, 37].

Databases included in this search are MEDLINE, Embase, Global Health, Social Policy and Practice, PsycINFO, Health Management Information Consortium, EBSCO CINAHL Plus, Cochrane Library and Web of Science. Searches will combine key terms relating to HIV (HIV, AIDS, human immune-deficiency syndrome, etc.) with terms related to self-testing (home-test, self-test, mail-test, home dried blood spot test, etc.). Five HIV conference databases (British HIV/AIDS Association, Conference on Retroviruses and Opportunistic Infections, European AIDS Society Conference, International AIDS Society and US National HIV Prevention Conference) will be systematically searched from January 2006 to the present date and then searched again annually as they become available. Additional grey literature search was not conducted. For full outline of database search strategy, see Additional file 2.

### Inclusion criteria

This systematic map will include peer-reviewed publications from a wide range of disciplines within social science and public health including epidemiology, sociology, anthropology, health economics and clinical practice. All study designs will be included. Examples include trials, observational data, economic evaluation and systematic reviews. Trials will include RCTs, cohort and other large-scale studies as well as smaller scale implementation, feasibility and demonstration projects. Observational studies will encompass quantitative epidemiological studies and cross-sectional studies of all types including qualitative, quantitative and mixed methods studies. Diagnostic accuracy studies will be coded as observational studies. Modelling will be included under the tag of economic evaluation. Systematic reviews and meta-analyses will be included.

Comments, reviews that are not systematic, opinion pieces and letters to the editor will be excluded (Table 1).

**Table 1:** Study codes and designs

Study types	Designs
Trials	RCTs and cohort studies
	Implementation, feasibility and demonstration projects
Observational studies	Epidemiological studies
	Cross-sectional studies (qualitative, quantitative and mixed methods)
	Diagnostic accuracy studies
Modelling	Economic evaluation
	Epidemiological modelling
Systematic reviews	Meta-analyses, meta-syntheses and meta-ethnography

Studies written in English and published after January 1, 2006, will be eligible for inclusion in this map. Grey literature beyond conference papers and abstracts will not be included as this map focuses on academic outputs which have been through peer-review processes.

### Screening

Eppi-Reviewer 4 will be used as a reference reviewing and management software for the duration of the project. Using this software, the titles and abstracts of studies only will be screened in duplicate against inclusion criteria by researchers from the study team and coded as EITHER eligible for inclusion OR ineligible, or THEY will be put forward for A second opinion. If it is not possible to ascertain whether a study is eligible based on title and abstract, it will be coded as requiring a second opinion and the full text will be obtained. Results from both researchers will be reconciled. Where disagreements exist, these will be resolved by full-text screening by both researchers. If disagreement persists, a third member of the study team will undertake a full-text review and provide an opinion.

### Data extraction and relational mapping

Evidence related to HIVST and HIVSS will be input into HIVST.org, a resource which consolidates and maps evidence and policy globally. Following further development, HIVST.org will become a public facing, open access relational database for policy makers, practitioners and academics to search for up to date information on HIVST. Results will be displayed on a map and through a searchable database. This will be completed through the following steps:

Studies which are eligible for inclusion will be coded by study design, population, year of publication, HIVST type and approach in EPPI-Reviewer 4. Following this coding, all studies will undergo a data extraction process, whereby key data are extracted and input into a Google document spreadsheet with pre-defined fields which link with those of a publicly available systematic map. This will be conducted by either a member of Sigma Research or a team member at the HIV Unit at the WHO. Data extraction will be done from full text where available, with abstract utilised when manuscripts cannot be sourced. Data extracted will include region, target population, aims, date of research, research methodology, study design, sample size, key results and summary of findings. Extraction for each study will be conducted by one member of the team only. See Additional file 3 for data extraction template.

The Google document spreadsheet will then be uploaded into the relational map. The externally facing map will have filterability as a database from the public facing end, with broad meta-level tags for study design as well as more specific tags for population, setting, HIVST type and key themes to allow for easy retrieval of results. This will enable identification of studies by key theme and easy comparison and interpretation of results. The Google document spreadsheet which the data is input through will have greater filtering capability, but these will only be available to the study team.

### Quality appraisal

During the systematic mapping process, we do not plan to undertake a formal quality appraisal of studies. The key purpose of such a process is to explore the existence and characteristics of the evidence base of a topic rather than to make judgements about the quality of studies themselves [24]. When systematic reviews are conducted using the results of our systematic map, then quality appraisal will be completed as relevant to the review type and nature of the evidence base.

## Outcomes

A key outcome of this systematic map will be the ongoing population of HIVST.org with results. Further, when sufficient evidence that is significantly different from the already published reviews emerges, then a systematic review can be completed from the subset of literature that is relevant to that specific research question.

It remains to be seen when sufficient evidence will emerge concerning use and effectiveness of HIVST (and HIVSS), but the systematic mapping exercise will facilitate the refining of additional or alternate questions for a systematic review. The suitability of conducting a further review will be assessed qualitatively through identifying emerging trends in the published literature and in conjunction with experts at the WHO. Having pre-completed, screening processes will allow for a timelier compilation of a systematic review when it is relevant to undertake one.

The precise approach taken for systematic reviewing (i.e. full meta-analysis, meta-summary, meta-ethnography, etc.) will be determined by what is most appropriate given the research question selected and the scope and quality of the pertinent evidence base.

## Discussion

This open access rapid systematic map will enable easy access to up-to-date information for policy makers, practitioners, academics and others with an interest in HIVST. It will also formalise a current WHO endeavour and provide a greater degree of rigour to HIVST.org. Further, having searches and filtering already completed will allow for a more rapid completion of a systematic review on a relevant topic when the necessary data emerges.

### Strengths and limitations

The approach outlined in this protocol has key strengths and limitations.

The ability to rapidly capture and characterise emerging literature which can then be used for a variety of application for academics and policy makers is a key strength. The transparent process through which this is completed provides intellectual rigour. This approach also allows for easy contextualisation of results from individual studies amongst the diverse evidence base of HIVST and HIVSS.

The major limitation of this type of review is that quality of evidence is not assessed. This rapid systematic mapping therefore gives all results equal weight in terms of prominence, and lower quality studies are not distinguished from those conducted with more rigour. It is therefore up to the audience to critically appraise the evidence base themselves.

## Additional files

Additional file 1: PRISMA checklist. (PDF 130 kb)

Additional file 2: Search strategy. Searches updated after peer review. (DOCX 19 kb)

Additional file 3: Data extraction template. Template to be used to extract data and upload to map. (XLSX 9 kb)
