# Supplementary material for: Consolidating emerging evidence surrounding HIVST and HIVSS: a rapid systematic mapping protocol
Source: Syst Rev. 2017 Apr 5;6:72. doi: 10.1186/s13643-017-0452-4 (PMC5382504; doi:10.1186/s13643-017-0452-4)
Supplement: Supplementary file 2 — Search strategy. Searches updated after peer review. (DOCX 19 kb) [file 13643_2017_452_MOESM2_ESM.docx]

## Appendix one: search strategy

Consolidating emerging evidence surrounding HIV self-testing (HIVST) & HIV self-sampling (HIVSS)

Search strategies: updated after peer review

The following databases were searched:

- Ovid SP Medline (Epub Ahead of Print, In-Process & Other Non-Indexed Citations, Ovid MEDLINE(R) Daily and Ovid MEDLINE(R) 1946 to Present, searched on 14 October 2016
- OvidSP Embase 1974 to 2016 October 13, searched on 14 October 2016
- OvidSP Global Health 1910 to 2016 week 39, searched on 14 October 2016
- OvidSP Social Policy & Practice 201607, searched on 14 October 2016
- OvidSP PsycINFO 1806 to October week 1 2016, searched on 14 October 2016
- OvidSP HMIC 1979 to September 2016, searched on 14 October 2016
- OvidSP Econlit 1886 to September 2016, searched on 18 October 2016
- EBSCOhost CINAHL Plus, searched on 14 October 2016
- Wiley Cochrane Library, searched on 14 October 2016
- Thomson Reuters Web of Science Core Collection, searched on 17 October 2016
- Elsevier SCOPUS, searched on 17 October 2016

All searched were limited to English language only and to results published from 2006 to present. Complete search strategies for each database are provided below. A total of 8860 items were retrieved. After duplicates were removed, 3571 unique items were retrieved.

For each database, these searches were used to create a monthly alert which emailed new papers to Charles Witzel.

# Search strategies used

## Database: OvidSP MEDLINE

Numbers in brackets are the number of items retrieved.

1. exp HIV Infections/ (253181)
2. exp HIV/ (89960)
3. hiv.ti,ab. (268385)
4. "hiv1".ti,ab. (793)
5. "hiv2".ti,ab. (155)
6. “hiv type 1".ti,ab. (3775)
7. "hiv type 2".ti,ab. (182)
8. human immunodeficiency virus.ti,ab. (77583)
9. human immunedeficiency virus.ti,ab. (6)
10. human immuno-deficiency virus.ti,ab. (214)
11. human immune-deficiency virus.ti,ab. (304)
12. (human immun* adj3 deficiency virus).ti,ab. (523)
13. acquired immunodeficiency syndrome.ti,ab. (15302)
14. acquired immunedeficiency syndrome.ti,ab. (13)
15. acquired immuno-deficiency syndrome.ti,ab. (116)
16. acquired immune-deficiency syndrome.ti,ab. (5387)
17. (acquired immun* adj3 deficiency syndrome).ti,ab. (5542)
18. Sexually Transmitted Diseases, Viral/ (1313)
19. or/1-18 (358848)
20. 20 (sample adj1 collect*).ti,ab. (8260)
21. Dried Blood Spot Testing/ and (home* or remote or personal or self*).ti,ab. (49)
22. (dried blood spot adj3 (home* or remote or personal or self*)).ti,ab. (16)
23. (alternative adj3 test*).ti,ab. (4973)
24. (option* adj1 test*).ti,ab. (408)
25. ((Home* or self* or mail*) adj3 (collection* or sampl* or specimen* or test* or kit)).ti,ab. (14504)
26. or/20-25 (27987)
27. 19 and 26 (1399)
28. limit 27 to english language (1357)
29. limit 28 to yr="2006 -Current" (911)

## Database: OvidSP Embase

Numbers in brackets are the number of items retrieved

1. exp Human immunodeficiency virus/ (236134)
2. exp Human immunodeficiency virus infection/ (338249)
3. hiv.ti,ab. (318347)
4. "hiv1".ti,ab. (1244)
5. "hiv2".ti,ab. (230)
6. "hiv type 1".ti,ab. (3840)
7. "hiv type 2".ti,ab. (188)
8. human immunodeficiency virus.ti,ab. (81710)
9. human immunedeficiency virus.ti,ab. (24)
10. human immuno-deficiency virus.ti,ab. (258)
11. human immune-deficiency virus.ti,ab. (381)
12. (human immun* adj3 deficiency virus).ti,ab. (646)
13. acquired immunodeficiency syndrome.ti,ab. (15678)
14. acquired immunedeficiency syndrome.ti,ab. (22)
15. acquired immuno-deficiency syndrome.ti,ab. (136)
16. acquired immune-deficiency syndrome.ti,ab. (5665)
17. (acquired immun* adj3 deficiency syndrome).ti,ab. (5828)
18. or/1-17 (460612)
19. (sample adj1 collect*).ti,ab. (11106)
20. Dried Blood Spot Testing/ and (home* or remote or personal or self*).ti,ab. (214)
21. (dried blood spot adj3 (home* or remote or personal or self*)).ti,ab. (21)
22. (alternative adj3 test*).ti,ab. (6145)
23. (option* adj1 test*).ti,ab. (569)
24. ((Home* or self* or mail*) adj3 (collection* or sampl* or specimen* or test* or kit)).ti,ab. (18541)
25. or/19-24 (36241)
26. 18 and 25 (1838)
27. limit 26 to english language (1789)
28. limit 27 to yr="2006 -Current" (1337)

## Database: OvidSP Global Health

Numbers in brackets are the number of items retrieved

1. exp human immunodeficiency viruses/ (151887)
2. hiv.ti,ab. (129335)
3. "hiv1".ti,ab. (252)
4. "hiv2".ti,ab. (36)
5. "hiv type 1".ti,ab. (2139)
6. "hiv type 2".ti,ab. (98)
7. human immunodeficiency virus.ti,ab. (36609)
8. human immunedeficiency virus.ti,ab. (3)
9. human immuno-deficiency virus.ti,ab. (71)
10. human immune-deficiency virus.ti,ab. (139)
11. (human immun* adj3 deficiency virus).ti,ab. (210)
12. acquired immunodeficiency syndrome.ti,ab. (4538)
13. acquired immunedeficiency syndrome.ti,ab. (4)
14. acquired immuno-deficiency syndrome.ti,ab. (39)
15. acquired immune-deficiency syndrome.ti,ab. (3398)
16. (acquired immun* adj3 deficiency syndrome).ti,ab. (3443)
17. or/2-17 (163755)
18. (sample adj1 collect*).ti,ab. (2272)
19. (dried blood spot adj3 (home* or remote or personal or self*)).ti,ab. (6)
20. (alternative adj3 test*).ti,ab. (851)
21. (option* adj1 test*).ti,ab. (83)
22. ((Home* or self* or mail*) adj3 (collection* or sampl* or specimen* or test* or kit)).ti,ab. (3760)
23. or/19-23 (6873)
24. 18 and 24 (749)
25. limit 25 to english language (715)
26. limit 26 to yr="2006 -Current" (563)

## Database: OvidSP Social Policy and Practice

Numbers in brackets are the number of items retrieved

1. hiv.ti,ab. (4096)
2. "hiv1".ti,ab. (2)
3. "hiv2".ti,ab. (0)
4. "hiv type 1".ti,ab. (1)
5. "hiv type 2".ti,ab. (0)
6. human immunodeficiency virus.ti,ab. (68)
7. human immunedeficiency virus.ti,ab. (0)
8. human immuno-deficiency virus.ti,ab. (4)
9. human immune-deficiency virus.ti,ab. (2)
10. (human immun* adj3 deficiency virus).ti,ab. (6)
11. acquired immunodeficiency syndrome.ti,ab. (19)
12. acquired immunedeficiency syndrome.ti,ab. (0)
13. acquired immuno-deficiency syndrome.ti,ab. (0)
14. acquired immune-deficiency syndrome.ti,ab. (19)
15. (acquired immun* adj3 deficiency syndrome).ti,ab. (19)
16. or/1-15 (4116)
17. (sample adj1 collect*).ti,ab. (8)
18. (dried blood spot adj3 (home* or remote or personal or self*)).ti,ab. (0)
19. (alternative adj3 test*).ti,ab. (24)
20. (option* adj1 test*).ti,ab. (3)
21. ((Home* or self* or mail*) adj3 (collection* or sampl* or specimen* or test* or kit)).ti,ab. (455)
22. or/17-21 (488)
23. 16 and 22 (11)

## Database: OvidSP PsycINFO

Numbers in brackets are the number of items retrieved

1. HIV testing/ (1916)
2. exp HIV/ (36936)
3. hiv.ti,ab. (42881)
4. "hiv1".ti,ab. (22)
5. "hiv2".ti,ab. (6)
6. "hiv type 1".ti,ab. (57)
7. "hiv type 2".ti,ab. (1)
8. human immunodeficiency virus.ti,ab. (5093)
9. human immunedeficiency virus.ti,ab. (1)
10. human immuno-deficiency virus.ti,ab. (12)
11. human immune-deficiency virus.ti,ab. (25)
12. (human immun* adj3 deficiency virus).ti,ab. (38)
13. acquired immunodeficiency syndrome.ti,ab. (669)
14. acquired immunedeficiency syndrome.ti,ab. (0)
15. acquired immuno-deficiency syndrome.ti,ab. (4)
16. acquired immune-deficiency syndrome.ti,ab. (2866)
17. (acquired immun* adj3 deficiency syndrome).ti,ab. (2873)
18. or/1-17 (47745)
19. (sample adj1 collect*).ti,ab. (436)
20. (dried blood spot adj3 (home* or remote or personal or self*)).ti,ab. (3)
21. (alternative adj3 test*).ti,ab. (1972)
22. (option* adj1 test*).ti,ab. (108)
23. ((Home* or self* or mail*) adj3 (collection* or sampl* or specimen* or test* or kit)).ti,ab. (10008)
24. or/19-23 (12484)
25. 18 and 24 (382)
26. limit 25 to english language (367)
27. limit 26 to yr="2006 -Current" (270)

## Database: Ovid SP HMIC Health Management Information Consortium

Numbers in brackets are the number of items retrieved

1. hiv tests/ (108)
2. HIV/ (3490)
3. acquired immune deficiency syndrome/ (3178)
4. hiv.ti,ab. (4302)
5. "hiv1".ti,ab. (2)
6. "hiv2".ti,ab. (0)
7. "hiv type 1".ti,ab. (0)
8. "hiv type 2".ti,ab. (0)
9. human immunodeficiency virus.ti,ab. (607)
10. human immunedeficiency virus.ti,ab. (0)
11. human immuno-deficiency virus.ti,ab. (4)
12. human immune-deficiency virus.ti,ab. (24)
13. (human immun* adj3 deficiency virus).ti,ab. (28)
14. acquired immunodeficiency syndrome.ti,ab. (114)
15. acquired immunedeficiency syndrome.ti,ab. (0)
16. acquired immuno-deficiency syndrome.ti,ab. (1)
17. acquired immune-deficiency syndrome.ti,ab. (181)
18. (acquired immun* adj3 deficiency syndrome).ti,ab. (182)
19. or/1-18 (6027)
20. (sample adj1 collect*).ti,ab. (13)
21. (dried blood spot adj3 (home* or remote or personal or self*)).ti,ab. (0)
22. (alternative adj3 test*).ti,ab. (41)
23. (option* adj1 test*).ti,ab. (9)
24. ((Home* or self* or mail*) adj3 (collection* or sampl* or specimen* or test* or kit)).ti,ab. (375)
25. or/20-24 (438)
26. 19 and 25 (17)
27. limit 26 to yr="2006 -Current" (9)

## Database: OvidSP EconLit

Numbers in brackets are the number of items retrieved

1. hiv.ti,ab. (1630)
2. "hiv1".ti,ab. (0)
3. "hiv2".ti,ab. (0)
4. "hiv type 1".ti,ab. (0)
5. "hiv type 2".ti,ab. (0)
6. human immunodeficiency virus.ti,ab. (70)
7. human immunedeficiency virus.ti,ab. (0)
8. human immuno-deficiency virus.ti,ab. (0)
9. human immune-deficiency virus.ti,ab. (0)
10. (human immun* adj3 deficiency virus).ti,ab. (0)
11. acquired immunodeficiency syndrome.ti,ab. (8)
12. acquired immunedeficiency syndrome.ti,ab. (0)
13. acquired immuno-deficiency syndrome.ti,ab. (2)
14. acquired immune-deficiency syndrome.ti,ab. (24)
15. (acquired immun* adj3 deficiency syndrome).ti,ab. (26)
16. or/1-15 (1664)
17. (sample adj1 collect*).ti,ab. (59)
18. (dried blood spot adj3 (home* or remote or personal or self*)).ti,ab. (0)
19. (alternative adj3 test*).ti,ab. (1065)
20. (option* adj1 test*).ti,ab. (17)
21. ((Home* or self* or mail*) adj3 (collection* or sampl* or specimen* or test* or kit)).ti,ab. (398)
22. or/17-21 (1538)
23. 16 and 22 (2)

## Database: EBSCO CINAHL Plus with Full Text

Numbers in brackets are the number of items retrieved

S1. (MH "HIV Infections+") (67,057)
S2. (MH "Human Immunodeficiency Virus+") (6,368)
S3. (TI hiv) OR (AB hiv) (61,304)
S4. (TI hiv1) OR (AB hiv1) (3,838)
S5. (TI hiv2) or (AB hiv2) (175)
S6. (TI hiv type 1) or (AB hiv type 1) (624)
S7. (TI hiv type 2) or (AB hiv type 2) (13)
S8. (TI human immunodeficiency virus) or (AB human immunodeficiency virus) (35,129)
S9. (TI human immunedeficiency virus) OR (AB human immunedeficiency virus) (0)
S10. (TI human immuno-deficiency virus) or (AB human immuno-deficiency virus) (19)
S11. (TI human immune-deficiency virus) OR (AB human immune-deficiency virus) (30)
S12. human immun* N3 deficiency virus (49)
S13. (TI acquired immunodeficiency syndrome) or (AB acquired immunodeficiency syndrome) (1,911)
S14. (TI acquired immunedeficiency syndrome) or (AB acquired immunedeficiency syndrome) (1)
S15. (TI acquired immuno-deficiency syndrome) OR (AB acquired immuno-deficiency syndrome) (1,655)
S16. (TI acquired immune-deficiency syndrome) or (AB acquired immune-deficiency syndrome) (1,785)
S17. acquired immun* N3 deficiency syndrome (662)
S18. S1 OR S2 OR S3 OR S4 OR S5 OR S6 OR S7 OR S8 OR S9 OR S10 OR S11 OR S12 OR S13 OR S14 OR S15 OR S16 OR S17 (85,288)
S19. (MH "Home Diagnostic Tests") (511)
S20. sample N1 collect* (6,919)
S21. (TI dried blood spot N3 (home* or remote or personal or self*)) or (AB dried blood spot N3 (home* or remote or personal or self*)) (7)
S22. alternative N3 test* (777)
S23. option* N1 test* (182)
S24. (home* N3 collection*) OR (home* N3 sampl*) or (home* N3 specimen*) or (home* N3 test*) or (home* N3 kit) or (self* N3 collection*) or (self* N3 sampl*) or (self* N3 specimen*) or (self* N3 test*) or (self* N3 kit) or (mail* N3 collection*) or (mail* N3 sampl*) or (mail* N3 specimen*) or (mail* N3 test*) or (mail* N3 kit) (7,399)
S25. S19 OR S20 OR S21 OR S22 OR S23 OR S24 (15,096)
S26. S18 AND S25 (766)
S27. S26 Limiters – English Language (762)
S28. S27 Limiters – Published date: 20060101 – (561)

## Database: Cochrane Library

Numbers in brackets are the number of items retrieved

#1. MeSH descriptor: [HIV Infections] explode all trees (9156)
#2. MeSH descriptor: [HIV] explode all trees (2885)
#3. hiv:ti,ab,kw (Word variations have been searched) (14031)
#4. "hiv1":ti,ab,kw (Word variations have been searched) (14)
#5. "hiv2":ti,ab,kw (Word variations have been searched) (0)
#6. "hiv type 1":ti,ab,kw (Word variations have been searched) (191)
#7. "hiv type 2":ti,ab,kw (Word variations have been searched) (0)
#8. "human immunodeficiency virus":ti,ab,kw (Word variations have been searched) (5774)
#9. "human immunedeficiency virus":ti,ab,kw (Word variations have been searched) (0)
#10. "human immuno-deficiency virus":ti,ab,kw (Word variations have been searched) (9)
#11. "human immune-deficiency virus":ti,ab,kw (Word variations have been searched) (3)
#12. "human immun*" near/3 "deficiency virus":ti,ab,kw (Word variations have been searched) (12)
#13. "acquired immunodeficiency syndrome":ti,ab,kw (Word variations have been searched) (1494)
#14. "acquired immunedeficiency syndrome":ti,ab,kw (Word variations have been searched) (1)
#15. "acquired immuno-deficiency syndrome":ti,ab,kw (Word variations have been searched) (2)
#16. "acquired immune-deficiency syndrome":ti,ab,kw (Word variations have been searched) (562)
#17. "acquired immun*" near/3 "deficiency syndrome":ti,ab,kw (Word variations have been searched) (566)
#18. MeSH descriptor: [Sexually Transmitted Diseases, Viral] this term only (25)
#19. #1 or #2 or #3 or #4 or #5 or #6 or #7 or #8 or #9 or #10 or #11 or #12 or #13 or #14 or #15 or #16 or #17 or #18 (15086)
#20. sample near/1 collect*:ti,ab,kw (Word variations have been searched) (1438)
#21. ("dried blood spot" near/3 home*) or ("dried blood spot" near/3 remote) or ("dried blood spot" near/3 personal) or ("dried blood spot" near/3 self*):ti,ab,kw (Word variations have been searched) (2)
#22. alternative near/3 test*:ti,ab,kw (Word variations have been searched) (257)
#23. option* near/1 test*:ti,ab,kw (Word variations have been searched) (20)
#24. (home* near/3 collection*) or (home* near/3 sampl*) or (home* near/3 specimen*) or (home* near/3 test*) or (home* near/3 kit) or (self* near/3 collection*) or (self* near/3 sampl*) or (self* near/3 specimen*) or (self* near/3 test*) or (self* near/3 kit) or (mail* near/3 collection*) or (mail* near/3 sampl*) or (mail* near/3 specimen*) or (mail* near/3 test*) or (mail* near/3 kit):ti,ab,kw (Word variations have been searched) (2503)
#25. #20 or #21 or #22 or #23 or #24 (4161)
#26. #19 and #25 (216)

This breaks down as:

- Cochrane Reviews (8)
- Other Reviews (1)
- Trials (201)
- Methods Studies (6)
- Technology Assessments (0)
- Economic Evaluations (0)
- Cochrane Groups (0)

## Database: Web of Science™ Core Collection

Numbers in brackets are the number of items retrieved. All searches run across Indexes = SCI-EXPANDED, SSCI, A&HCI, CPCI-S, CPCI-SSH, ESCI. Timespan = 2006-2016

#1. TOPIC: (hiv) (162,466)
#2. TOPIC: (hiv1) (338)
#3. TOPIC: (hiv2) (32)
#4. TOPIC: ("hiv type 1") (1,561)
#5. TOPIC: ("hiv type 2") (50)
#6. TOPIC: ("human immunodeficiency virus") (43,584)
#7. TOPIC: ("human immunedeficiency virus") (4)
#8. TOPIC: ("human immuno-deficiency virus") (80)
#9. TOPIC: ("human immune-deficiency virus") (119)
#10. TOPIC: ("human immun*" NEAR/3 "deficiency virus") (202)
#11. TOPIC: ("acquired immunodeficiency syndrome") (4,393)
#12. TOPIC: ("acquired immunedeficiency syndrome") (1)
#13. TOPIC: ("acquired immuno-deficiency syndrome") (31)
#14. TOPIC: ("acquired immune-deficiency syndrome") (1,046)
#15. #14 OR #13 OR #12 OR #11 OR #10 OR #9 OR #8 OR #7 OR #6 OR #5 OR #4 OR #3 OR #2 OR #1 (170,925)
#16. TOPIC: (sample NEAR/1 collect*) (93,366)
#17. TOPIC: ("dried blood spot" NEAR/3 home*) (10)
#18. TOPIC: ("dried blood spot" NEAR/3 remote) (1)
#19. TOPIC: ("dried blood spot" NEAR/3 personal) (2)
#20. TOPIC: ("dried blood spot" NEAR/3 self*) (7)
#21. TOPIC: (alternative NEAR/3 test*) (7,486)
#22. TOPIC: (option* NEAR/1 test*) (710)
#23. TOPIC: (home* NEAR/3 collection*) (308)
#24. TOPIC: (home* NEAR/3 sampl*) (1,878)
#25. TOPIC: (home* NEAR/3 specimen*) (66)
#26. TOPIC: (home* NEAR/3 test*) (2,607)
#27. TOPIC: (home* NEAR/3 kit) (198)
#28. TOPIC: (self* NEAR/3 collection*) (652)
#29. TOPIC: (self* NEAR/3 sampl*) (3,407)
#30. TOPIC: (self* NEAR/3 specimen*) (405)
#31. TOPIC: (self* NEAR/3 test*) (8,991)
#32. TOPIC: (self* NEAR/3 kit) (200)
#33. TOPIC: (mail* NEAR/3 collection*) (79)
#34. TOPIC: (mail* NEAR/3 sampl*) (577)
#35. TOPIC: (mail* NEAR/3 specimen*) (25)
#36. TOPIC: (mail* NEAR/3 test*) (227)
#37. TOPIC: (mail* NEAR/3 kit) (81)
#38. #37 OR #36 OR #35 OR #34 OR #33 OR #32 OR #31 OR #30 OR #29 OR #28 OR #27 OR #26 OR #25 OR #24 OR #23 OR #22 OR #21 OR #20 OR #19 OR #18 OR #17 OR #16 (119,346)
#39. #38 AND #15 (2,427)
#40. #38 AND #15 Refined by: LANGUAGES: ( ENGLISH ) (2,393)

## SCOPUS

Numbers in brackets are the number of items retrieved

( ( TITLE-ABS-KEY ( hiv ) ) OR ( TITLE-ABS-KEY ( "hiv1" ) ) OR ( TITLE-ABS-KEY ( "hiv2" ) ) OR ( TITLE-ABS-KEY ( "hiv type 1" ) ) OR ( TITLE-ABS-KEY ( "hiv type 2" ) ) OR ( TITLE-ABS-KEY ( "human immunodeficiency virus" ) ) OR ( TITLE-ABS-KEY ( "human immunedeficiency virus" ) ) OR ( TITLE-ABS-KEY ( "human immuno-deficiency virus" ) ) OR ( TITLE-ABS-KEY ( "human immune-deficiency virus" ) ) OR ( TITLE-ABS-KEY ( "human immun*" W/3 "deficiency virus" ) ) OR ( TITLE-ABS-KEY ( "acquired immunodeficiency syndrome" ) ) OR ( TITLE-ABS-KEY ( "acquired immunedeficiency syndrome" ) ) OR ( TITLE-ABS-KEY ( "acquired immuno-deficiency syndrome" ) ) OR ( TITLE-ABS-KEY ( "acquired immune-deficiency syndrome" ) ) OR ( TITLE-ABS-KEY ( "acquired immun*" W/3 "deficiency syndrome" ) ) ) AND ( ( TITLE-ABS-KEY ( sample W/1 collect* ) ) OR ( TITLE-ABS-KEY ( "dried blood spot" W/3 home* ) ) OR ( TITLE-ABS-KEY ( "dried blood spot" W/3 remote ) ) OR ( TITLE-ABS-KEY ( "dried blood spot" W/3 personal ) ) OR ( TITLE-ABS-KEY ( "dried blood spot" W/3 self* ) ) OR ( TITLE-ABS-KEY ( alternative W/3 test* ) ) OR ( TITLE-ABS-KEY ( option W/1 test* ) ) OR ( TITLE-ABS-KEY ( home* W/3 collection* ) ) OR ( TITLE-ABS-KEY ( home* W/3 sampl* ) ) OR ( TITLE-ABS-KEY ( home* W/3 specimen* ) ) OR ( TITLE-ABS-KEY ( home* W/3 test* ) ) OR ( TITLE-ABS-KEY ( home* W/3 kit ) ) OR ( TITLE-ABS-KEY ( self* W/3 collection* ) ) OR ( TITLE-ABS-KEY ( self* W/3 sampl* ) ) OR ( TITLE-ABS-KEY ( self* W/3 specimen* ) ) OR ( TITLE-ABS-KEY ( self* W/3 test* ) ) OR ( TITLE-ABS-KEY ( self* W/3 kit ) ) OR ( TITLE-ABS-KEY ( mail* W/3 collection* ) ) OR ( TITLE-ABS-KEY ( mail* W/3 sampl* ) ) OR ( TITLE-ABS-KEY ( mail* W/3 specimen* ) ) OR ( TITLE-ABS-KEY ( mail* W/3 test* ) ) OR ( TITLE-ABS-KEY ( mail* W/3 kit ) ) ) AND ( LIMIT-TO ( PUBYEAR , 2017 ) OR LIMIT-TO ( PUBYEAR , 2016 ) OR LIMIT-TO ( PUBYEAR , 2015 ) OR LIMIT-TO ( PUBYEAR , 2014 ) OR LIMIT-TO ( PUBYEAR , 2013 ) OR LIMIT-TO ( PUBYEAR , 2012 ) OR LIMIT-TO ( PUBYEAR , 2011 ) OR LIMIT-TO ( PUBYEAR , 2010 ) OR LIMIT-TO ( PUBYEAR , 2009 ) OR LIMIT-TO ( PUBYEAR , 2008 ) OR LIMIT-TO ( PUBYEAR , 2007 ) OR LIMIT-TO ( PUBYEAR , 2006 ) ) AND ( LIMIT-TO ( LANGUAGE , "English" ) ) (2587)
